# 

**Published:** 1881-11

**Authors:** 


### CONTENTS.

| | Page. |
|---|---|
| Contagious Diseases | 401 |
| Anæmia | 403 |
| Don't take it to Heart (poetry) | 404 |
| The Case of President Garfield | 405 |
| Anti-Monopoly | 406 |
| Reserved Force | 407 |
| Importance of Mineral Elements of Food | 408 |
| Concerning Swimming | 411 |
| The End of a Fortune | 412 |
| The Utilities | 413 |
| Throat-Ail | 415 |
| Popular Fallacies | 425 |
| Flower Pots | 426 |
| Reason in all Things | 427 |
| Shaving the Beard | 428 |
| The Manners of Nations | 429 |
| How Sponges are Caught | 430 |
| To Sweep and Dust | 431 |
| A Cure for Small-Pox | 432 |
| Whence They Came | 432 |
| Words of Courage | 434 |
| Items | 434 |
| How to Keep Your Friends | 436 |
| Sanitary Department of the Journal | 437 |

